# FSC-BiMamba: A Dual-Branch Bidirectional Mamba Network for Frontal Sparse-Channel EEG Depression Detection

**DOI:** 10.3390/brainsci16090902

**Published:** 2026-08-24

**Authors:** Yaqiang Che, Chunting Wan, Wenhao Yang, Dongyi Chen

**Affiliations:** 1 Guangxi Key Laboratory of Automatic Detecting Technology and Instruments, School of Electronic Engineering and Automation, Guilin University of Electronic Technology, Guilin 541004, China; cheyaqiang@mails.guet.edu.cn (Y.C.); ywh@mails.guet.edu.cn (W.Y.); 2 School of Automation Engineering, University of Electronic Science and Technology of China, Chengdu 611731, China; dychen@uestc.edu.cn

**Keywords:** major depressive disorder, sparse-channel electroencephalogram, subject-independent window-level classification, bidirectional Mamba, deep learning

## Abstract

**Background**: Major depressive disorder (MDD) is a common psychiatric disorder. Electroencephalography (EEG) provides physiological information for depression detection, but many existing methods rely on dense multichannel recordings, limiting their use in lightweight EEG screening. Frontal sparse-channel EEG reduces acquisition burden but provides limited spatial information, requiring effective within-window representation learning and cross-window temporal modelling. **Methods**: We propose FSC-BiMamba, a dual-branch bidirectional Mamba network for subject-independent window-level MDD classification from frontal sparse-channel EEG. For each window, a Time–Frequency Map Encoder (TFME) learns local time–frequency patterns, while a Frequency-Domain Statistical Descriptor Encoder (FSDE) encodes complementary frequency-domain statistical descriptors. An Adaptive Dual-Token Fusion (ADTF) module integrates the resulting tokens through feature-wise gating to form a unified window representation. Representations from eight consecutive windows are then processed by a bidirectional Mamba (BiMamba) module to capture cross-window context. Finally, a softmax classifier converts each contextualized representation into a window-level class prediction. **Results**: Performance was evaluated using stratified subject-independent five-fold cross-validation. FSC-BiMamba achieved window-level accuracies of 82.70 ± 2.37% and 84.55 ± 8.01% on MODMA and Mumtaz2016, respectively. Together with its compact architecture, these results indicate a favourable balance between classification performance and model size. **Conclusions**: FSC-BiMamba effectively integrates complementary window-level representations with cross-window temporal context. It achieved the highest accuracy among the compared models on both datasets, demonstrating a favourable performance–size trade-off for lightweight EEG-based MDD screening.

## 1. Introduction

Major depressive disorder (MDD) is a leading contributor to the global burden of disease [1]. However, its clinical diagnosis still relies largely on symptom scales, interviews, and professional judgement, which may be affected by self-report bias, comorbidity, symptom fluctuation, and inter-rater variability. Electroencephalography (EEG) provides a non-invasive and temporally precise measure of brain activity, and previous studies have reported depression-related alterations in frontal alpha asymmetry, oscillatory dynamics, nonlinear characteristics, and functional connectivity [2]. Recent reviews have further shown that spectral, time–frequency, complexity, connectivity, and deep representations can provide complementary information for EEG-based depression detection [3,4,5]. These findings indicate that MDD-related EEG characteristics are distributed across multiple signal dimensions rather than being fully captured by a single type of feature.

Early EEG-based MDD detection studies mainly relied on handcrafted features and conventional machine-learning classifiers. Mohammadi et al. extracted linear and nonlinear EEG characteristics and demonstrated their diagnostic value using data-mining methods [6]. Liao et al. combined spectral and nonlinear features with machine learning to distinguish patients with MDD from healthy controls [7], while Hosseinifard et al. showed that nonlinear EEG descriptors could support depression classification [8]. These studies demonstrate that multiple handcrafted feature categories can provide useful diagnostic information. However, handcrafted pipelines depend heavily on feature design, channel configuration, and prior neurophysiological assumptions. Their robustness may therefore decrease when acquisition settings, subject distributions, or recording montages change.

Deep learning has been introduced to reduce dependence on manual feature engineering. Convolutional neural networks can learn local temporal, spectral, or spatial patterns directly from EEG data. Schirrmeister et al. showed that deep convolutional networks can learn interpretable EEG decoding patterns related to spectral power changes [9]. EEGNet then introduced a compact architecture based on temporal convolution, depthwise spatial filtering, and separable convolution, providing an efficient neural design for EEG decoding [10]. For EEG-based MDD detection, Rafiei et al. treated depression recognition as a time-series classification problem and used deep learning to learn discriminative EEG representations [11]. Transformer-related models have also been explored for EEG representation learning. EEG Conformer integrates convolutional feature extraction with self-attention for EEG decoding and visualization [12]. SLiTRANet combines local graph convolution and Transformer-based modelling for automated MDD monitoring [13]. More recently, Umair et al. proposed a decentralized EEG-based MDD detection framework using Transformer architectures and split learning [14]. These studies show a clear shift from purely feature-engineered classification toward learnable, structured, and deployment-aware EEG representation learning.

Despite these advances, many existing models still assume multi-channel EEG input. Multi-channel EEG provides rich spatial information and supports spatial filtering, functional connectivity matrices, graph neural networks, and spatial attention mechanisms. Zhang et al. constructed resting-state EEG functional brain networks for MDD analysis and classification, showing that inter-channel connectivity can provide discriminative information [15]. Jiang et al. improved EEG-based depression classification by incorporating spatial information, further demonstrating the importance of spatial distribution in multi-channel EEG modelling [16]. Chen et al. proposed MGSN, a lightweight depression EEG model based on multiscale dynamic graph convolution and spiking neural networks for multichannel topology modelling [17]. Xu et al. introduced TFAGL, which uses time–frequency EEG and agent graph learning for MDD detection [18]. Channel-relationship modelling has also been validated in affective EEG analysis. For example, CR-GCN uses graph convolution to model EEG channel relationships for emotion recognition [19]. Zhang et al. further proposed ACM-GNN, which adaptively constructs cluster-oriented brain network modularity and learns interactions between global-level and modular-level graph representations [20]. These studies highlight the value of topological and spatial modelling. However, such methods usually depend on sufficient electrode nodes, reliable inter-channel edges, and stable spatial layouts. In lightweight screening, wearable acquisition, and rapid-deployment scenarios, high-density EEG increases setup complexity, preparation time, and acquisition cost.

Sparse frontal EEG offers a more practical alternative because frontal regions are closely associated with emotional regulation and depression-related neurophysiological alterations. Nevertheless, reducing the number of electrodes also removes much of the spatial information used by conventional multichannel models. Systematic validation has demonstrated the potential of resting-state EEG for MDD detection [21], while single-channel and low-channel studies have shown that diagnostically relevant information can still be extracted from compact acquisition systems [22,23]. Depression-related alterations in frontal functional connectivity further support the clinical relevance of frontal EEG signals [24]. However, sparse-channel analysis should not be regarded simply as a reduced version of multichannel modelling. With limited spatial coverage, the model must extract richer information from temporal dynamics, frequency distributions, and cross-channel statistical dependencies.

These signal dimensions provide complementary descriptions of EEG activity. Time–frequency representations characterize transient oscillatory patterns and changes in spectral energy and have been widely employed in automated depression analysis [25]. Phase-based measures such as the phase-locking value quantify synchronization between signals [26], whereas the weighted phase-lag index reduces the influence of volume conduction and zero-lag coupling [27]. Covariance representations describe statistical dependencies among channels and can be analyzed on the Riemannian manifold of symmetric positive-definite matrices [28]. However, most existing methods process these representations independently or combine them through direct concatenation or fixed weighting. Such strategies cannot adaptively determine which representation is more informative for a given EEG segment.

Adaptive feature fusion provides a possible solution by learning input-dependent weights for different representations. Channel-attention mechanisms have demonstrated that feature responses can be recalibrated according to their relative importance [29]. Meanwhile, state-space models such as Mamba enable efficient long-sequence modelling through selective information propagation [30]. Bidirectional variants can further incorporate both forward and backward contextual information [31]. Mamba-based models have recently been applied to physiological signals, including EEG and electrocardiography, demonstrating their potential for long-range biomedical sequence modelling [32]. Nevertheless, the combination of adaptive representation fusion and bidirectional cross-window temporal modelling remains insufficiently explored for sparse frontal EEG-based MDD detection.

While these methodological advances improve EEG-based representation learning, EEG captures only one level of the complex biological processes associated with MDD. Recent multimodal research integrating five neuroimaging modalities with plasma proteomic data identified a depression-related brain–body covariation component associated with metabolic dysfunction [33]. This finding broadens the biological context in which EEG biomarkers should be interpreted: EEG characterizes neural dynamics, whereas a more comprehensive understanding of MDD may ultimately require the integration of neural and peripheral biological information. The present study addresses the focused methodological problem of extracting reliable neurophysiological representations from sparse frontal EEG, while integration with systemic biomarkers remains an important direction for future research.

Within this scope, three limitations remain in existing sparse EEG-based MDD detection methods. First, they do not fully exploit the complementary information contained in time–frequency patterns, phase synchronization, and covariance geometry. Second, most fusion strategies are static and cannot adapt the contribution of different representations to individual EEG segments. Third, segment-wise classification neglects temporal dependencies across consecutive windows, even though resting-state EEG exhibits meaningful short- and medium-range fluctuations. Addressing these limitations requires a framework that can extract complementary features, adaptively fuse them, and model their temporal evolution without relying on dense electrode configurations.

To address these limitations, this study proposes FSC-BiMamba, a dual-branch bidirectional Mamba network for frontal sparse-channel EEG MDD detection. The main contributions are summarized as follows:

1. A dual-branch bidirectional Mamba framework called FSC-BiMamba is proposed for frontal sparse-channel EEG-based MDD detection. The framework integrates window-level representation learning with cross-window temporal modelling, making it suitable for information-limited EEG settings.
2. A Time–Frequency Map Encoder (TFME) is designed to learn local oscillatory patterns from Morlet time–frequency maps of short EEG windows. In parallel, a Frequency-Domain Statistical Descriptor Encoder (FSDE) is proposed to encode explicit EEG descriptors, including band power, bipolar power, asymmetry, spectral coupling, and log-covariance, into statistical tokens.
3. An Adaptive Dual-Token Fusion (ADTF) module and a BiMamba temporal modelling module are introduced. ADTF adaptively fuses learned time–frequency tokens and statistical descriptor tokens, whereas BiMamba captures bidirectional dependencies across consecutive EEG windows.
4. Extensive experiments are conducted on two public resting-state EEG datasets using subject-independent five-fold cross-validation. The results demonstrate the effectiveness of FSC-BiMamba for subject-independent MDD detection under frontal sparse-channel EEG settings.

The remainder of this paper is organized as follows. Section 2 presents the proposed FSC-BiMamba framework and its main modules. Section 3 describes the datasets, preprocessing procedures, validation strategy, and implementation details, and also reports the experimental results and analyses. A discussion of the results is given in Section 4. Section 5 concludes the paper.

## 2. Methods

### 2.1. Overview of FSC-BiMamba

**Figure 1:**
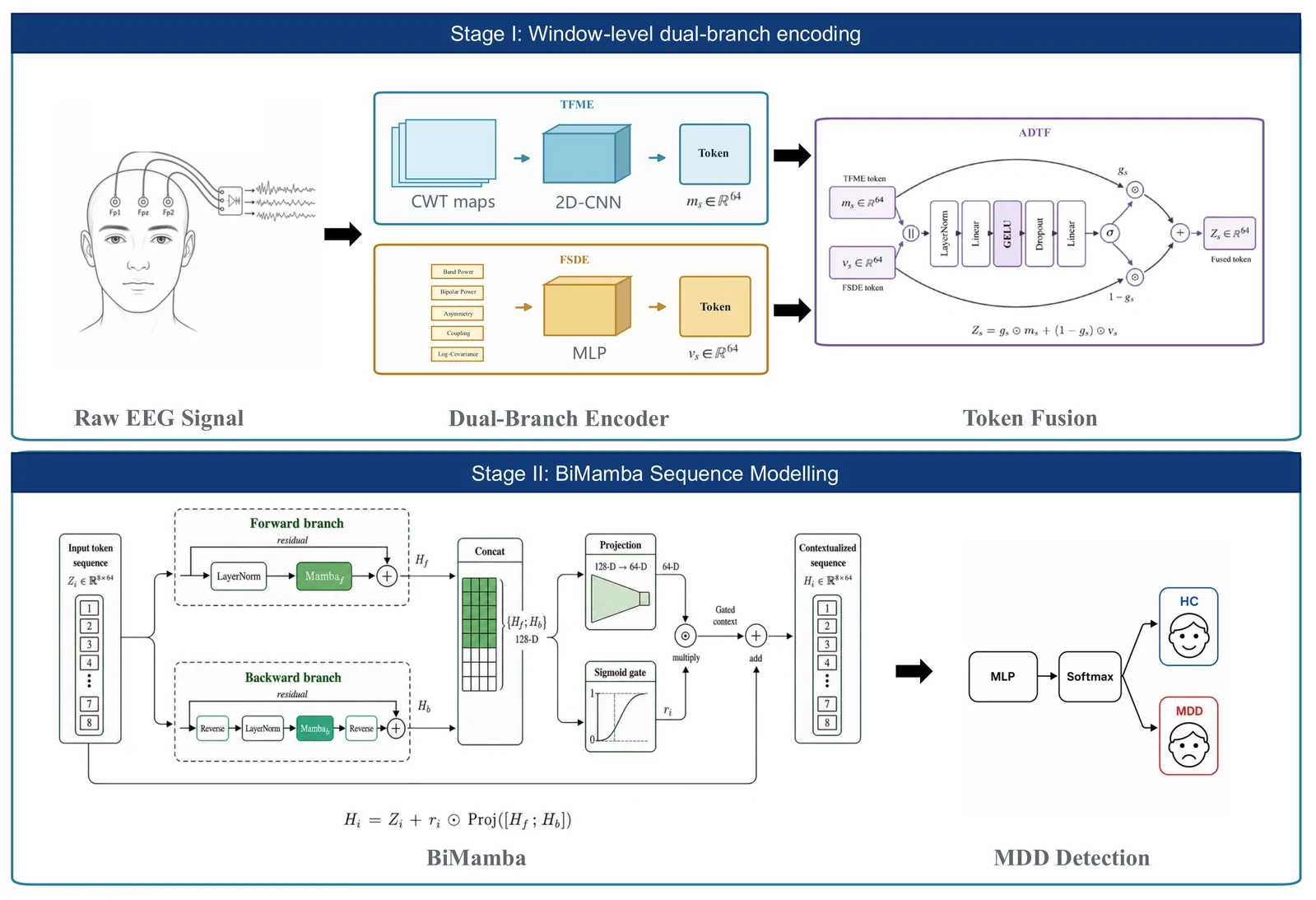
Overall framework of FSC-BiMamba.

We propose FSC-BiMamba, a dual-branch bidirectional Mamba network for frontal sparse-channel EEG depression detection. The model is designed to learn discriminative information from both individual EEG windows and their temporal context across consecutive windows. It consists of four core modules: the Time–Frequency Map Encoder (TFME), the Frequency-Domain Statistical Descriptor Encoder (FSDE), the Adaptive Dual-Token Fusion (ADTF) module and the BiMamba module. As shown in Figure 1, these four functional modules are organized into two successive stages: window-level encoding and sequence modelling.

During window-level encoding, TFME and FSDE process the same EEG window in parallel. TFME learns local oscillatory patterns from time–frequency maps, whereas FSDE encodes explicit frequency-domain statistics related to spectral power, inter-channel differences, functional coupling and covariance structure. ADTF then determines the feature-wise contributions of the two representations and produces a unified token for each window. During sequence modelling, the tokens of consecutive windows are processed by BiMamba in both temporal directions. This design allows FSC-BiMamba to integrate local time–frequency patterns, explicit frequency-domain descriptors and cross-window temporal dependencies within a unified framework.

The following subsections describe the proposed FSC-BiMamba in detail.

### 2.2. Window and Sequence Construction

Each continuous EEG recording was segmented into non-overlapping windows of duration $\tau_{w}$. Let $f_{s}$ denote the post-preprocessing sampling rate, C the number of input channels, and $T=f_{s}\tau_{w}$ the number of samples per window. The s-th EEG window is denoted as $x_{s}\in R^{C\times T}$. In this study, $f_{s}=250$ Hz, $\tau_{w}=2$ s, and C=3, yielding T=500 samples per window.

To enable cross-window modelling, S consecutive windows from the same participant and recording were arranged chronologically as $X=\left[x_{1},x_{2},\ldots ,x_{S}\right]\in R^{S\times C\times T}$. We set S=8, corresponding to a sequence duration of $S\tau_{w}=16$ s.

### 2.3. Time–Frequency Map Encoder

For the s-th EEG window, we denote the input as $x_{s}\in R^{C\times T}$. The Time–Frequency Map Encoder (TFME) first transforms each channel into a time–frequency representation using Morlet continuous wavelet transform:

(1) $$W_{s,c}\left(f,\tau \right)=\sum_{t=1}^{T}x_{s,c}\left(t\right)\psi_{f}^{*}\left(t-\tau \right),$$

where $\psi_{f}\left(\cdot \right)$ is the wavelet kernel at frequency f. The log-power time–frequency map is computed as

(2) $$P_{s,c}\left(f,\tau \right)=\log\left(1+{\left|W_{s,c}\left(f,\tau \right)\right|}^{2}\right).$$

The resulting maps are pooled into F frequency bins and L temporal bins. In the current implementation, F=45 and L=32. To adaptively reweight the three channels, TFME computes a channel attention vector from band-power statistics:

(3) $$\alpha_{s,c}=\mathrm{softmax}\left({\mathrm{MLP}}_{a}\left(b_{s}\right)\right),$$

where $b_{s}$ denotes the band-power summary of the current window. The channel-weighted map is

(4) $${\bar{P}}_{s,c}=\alpha_{s,c}P_{s,c}.$$

The enhanced time–frequency maps ${\bar{P}}_{s,c}$ are subsequently fed into a lightweight two-dimensional convolutional encoder. The encoder extracts local time–frequency patterns jointly along the temporal and frequency dimensions, producing a feature tensor $U_{s}\in R^{D\times F\times L}$, where D=64 denotes the feature dimension, and F and L represent the frequency and temporal dimensions, respectively. Band-aware aggregation is then applied to the encoded features. For the k-th EEG frequency band $\Omega_{k}$, the corresponding band token is defined as

(5) $$r_{s,k}=\frac{1}{\left|\Omega_{k}\right|L}\sum_{f\in \Omega_{k}}\sum_{\tau =1}^{L}U_{s}\left(:,f,\tau \right).$$

The attention weight of each band is calculated as

(6) $$\beta_{s,k}={\mathrm{softmax}}_{k}\left({\mathrm{MLP}}_{b}\left(r_{s,k}\right)\right).$$

The final TFME token is

(7) $$m_{s}=\sum_{k=1}^{K}\beta_{s,k}r_{s,k},$$

where $m_{s}\in R^{D}$. This token provides a compact representation of local time–frequency patterns in each EEG window.

### 2.4. Frequency-Domain Statistical Descriptor Encoder

The Frequency-Domain Statistical Descriptor Encoder (FSDE) complements the map-based features learned by TFME by extracting explicit frequency-domain statistical descriptors. For each EEG window $x_{s}\in R^{C\times T}$, the spectrum is divided into five predefined EEG bands: δ (1–4 Hz), θ (4–8 Hz), α (8–13 Hz), β (13–30 Hz), and γ (30–45 Hz). Within these bands, FSDE computes original-channel band power, bipolar band power, inter-channel asymmetry, spectral coupling, and band-wise log-covariance descriptors. These descriptor groups contain 15, 15, 20, 60, and 30 features, respectively, and are concatenated into a P-dimensional descriptor, where P=140 in this study. A two-layer multilayer perceptron maps this descriptor to a D-dimensional FSDE token $v_{s}\in R^{D}$, with D=64 in the current implementation. In contrast to TFME, which learns local patterns from time–frequency maps, FSDE explicitly preserves spectral energy distributions, inter-channel differences, pairwise coupling, and multichannel covariance structure.

For spectral-power estimation, the discrete Fourier transform is first applied to Fp1, Fpz and Fp2. Let $X_{s,c}\left(f\right)$ denote the Fourier coefficient of channel c at frequency f. The logarithmic power of channel c in the k-th frequency band $\Omega_{k}$ is calculated as

(8) $$p_{s,c,k}=\log\left(1+\frac{1}{\left|\Omega_{k}\right|}\sum_{f\in \Omega_{k}}{\left|X_{s,c}\left(f\right)\right|}^{2}\right).$$

The five-band powers of the three original channels produce 15 features. To describe local spectral differences, three bipolar signals, Fp1–Fpz, Fp2–Fpz and Fp1–Fp2, are constructed in the time domain and processed using the same procedure, yielding another 15 features. Original-channel powers characterize the spectral-energy distribution at individual electrodes, whereas bipolar powers emphasize relative activity between neighbouring channels and reduce components shared by both electrodes.

FSDE then derives asymmetry descriptors from the original-channel band powers. For each frequency band, the left–right difference is obtained from Fp1 and Fp2, and its normalized form is defined as

(9) $$a_{s,k}^{LR}=\frac{p_{s,Fp2,k}-p_{s,Fp1,k}}{\left|p_{s,Fp2,k}\right|+\left|p_{s,Fp1,k}\right|+\varepsilon }.$$

In addition to the raw and normalized left–right differences, FSDE calculates the difference between Fpz and the average of Fp1 and Fp2, together with its normalized counterpart. These four descriptors are computed in each of the five frequency bands, producing 20 asymmetry features. The unnormalized differences retain the magnitude of inter-channel deviations, whereas the normalized descriptors reduce their dependence on the overall signal scale.

To characterize pairwise spectral coupling, FSDE considers the Fp1–Fpz, Fpz–Fp2 and Fp1–Fp2 channel pairs. For channels i and j, the normalized cross-spectrum within band $\Omega_{k}$ is calculated as

(10) $$\rho_{s,ij,k}=\frac{\sum_{f\in \Omega_{k}}X_{s,i}\left(f\right)X_{s,j}^{*}\left(f\right)}{\sqrt{\left(\sum_{f\in \Omega_{k}}{\left|X_{s,i}\left(f\right)\right|}^{2}\right)\left(\sum_{f\in \Omega_{k}}{\left|X_{s,j}\left(f\right)\right|}^{2}\right)}+\varepsilon }.$$

The magnitude of $\rho_{s,ij,k}$ is retained as the coherence descriptor, while the magnitude of its imaginary component is used as imaginary coherence. FSDE additionally computes the phase-locking value from the circular mean of the within-band phase differences. These descriptors characterize complementary properties of amplitude-normalized and phase-based relationships between the three channels.

The weighted phase-lag index is further included to emphasize consistently signed non-zero-lag interactions. It is calculated from the imaginary component of the cross-spectrum as

(11) wPLIs,ij,k=|∑f∈ΩkIm{Xs,i(f)Xs,j∗(f)}|∑f∈Ωk|Im{Xs,i(f)Xs,j∗(f)}|+ε.

For each of the three channel pairs, coherence, imaginary coherence, phase-locking value and weighted phase-lag index are calculated in all five frequency bands. The resulting 3×4×5 measurements form 60 coupling descriptors. Together, these features describe whether the channels exhibit consistent spectral and phase relationships within the same EEG window.

Pairwise coupling measures describe individual channel pairs but do not fully characterize the joint variation of all three channels. FSDE therefore supplements them with band-wise covariance descriptors. For each frequency band, the Fourier coefficients outside the target band are masked, and an inverse Fourier transform reconstructs the corresponding band-limited three-channel signal. After temporal centring, a 3×3 covariance matrix $C_{s,k}$ is calculated and normalized by its mean diagonal value. A small identity matrix is added for numerical stability, after which the matrix logarithm is applied:

(12) $$L_{s,k}=\log_{m}\left(\frac{C_{s,k}}{\mathrm{tr}\left(C_{s,k}\right)/3}+\varepsilon I_{3}\right),$$

Here, $\log_{m}\left(\cdot \right)$ denotes the matrix logarithm. Because $L_{s,k}$ is symmetric, only its six upper-triangular elements are retained. Applying this procedure to five frequency bands produces 30 log-covariance features. This representation captures band-specific multichannel variation in a compact form while avoiding redundant symmetric elements.

Finally, the five descriptor groups are concatenated in a fixed order to form the FSDE descriptor. The descriptor is first normalized using layer normalization and then passed through a two-layer multilayer perceptron. The first linear layer maps the input into a hidden space, followed by GELU activation and dropout. The second linear layer produces the FSDE token $v_{s}\in R^{D}$, followed by GELU activation.

### 2.5. Adaptive Dual-Token Fusion Module

TFME and FSDE produce two tokens for the same EEG window: $m_{s}$ and $v_{s}$. Direct concatenation retains all features but increases the representation dimension and leaves the following layers to determine the contribution of each branch. Fixed averaging preserves the dimensionality but assumes that TFME and FSDE are equally informative for every window. ADTF instead performs feature-wise adaptive fusion.

The two D-dimensional tokens are concatenated and passed through a lightweight gating network consisting of layer normalization, two linear layers, GELU activation and dropout. A sigmoid activation produces a D-dimensional gate:

(13) $$g_{s}=\sigma \left({\mathrm{MLP}}_{\mathrm{ADTF}}\left(\left[m_{s};v_{s}\right]\right)\right).$$

The gate controls the contribution of the two branches independently in each feature dimension:

(14) $$z_{s}=g_{s}⊙m_{s}+\left(1-g_{s}\right)⊙v_{s},\;\;z_{s}\in R^{D}.$$

When an element of $g_{s}$ approaches one, the corresponding TFME feature receives greater weight; when it approaches zero, the FSDE feature dominates. Layer normalization and dropout are applied after fusion to obtain the final window token passed to BiMamba. ADTF therefore preserves the D-dimensional token size while allowing the composition of the representation to vary across windows.

### 2.6. BiMamba Module

The window-level encoder is shared across all positions in a sequence. After the S windows have been independently encoded and fused, their tokens are arranged chronologically to form $Z_{i}\in R^{S\times D}$.

The BiMamba module models this sequence in both temporal directions. The forward sequence representation is

(15) $$H_{f}={\mathrm{Mamba}}_{f}\left(Z\right).$$

The backward sequence representation is

(16) $$H_{b}=\mathrm{Reverse}\left({\mathrm{Mamba}}_{b}\left(\mathrm{Reverse}\left(Z\right)\right)\right).$$

Each directional block applies layer normalization before the sequence operator and adds its output to the input through a residual connection. The forward representation contains information accumulated from preceding windows, whereas the backward representation provides context from subsequent windows. The two directional representations are concatenated at each window position. A projection path maps the 2D-dimensional concatenated representation back to D dimensions, while a parallel sigmoid gate determines how much bidirectional context should be introduced. The contextualized sequence is obtained as

(17) $$H_{i}=Z_{i}+r_{i}⊙\mathrm{Proj}\left(\left[H_{f};H_{b}\right]\right),$$

where $r_{i}$ is the gate generated from the concatenated forward and backward features. The residual connection preserves the original window representation, while the gate suppresses contextual information that is not useful for a particular window or feature dimension.

### 2.7. MDD Detection

The contextualized sequence $H_{i}\in R^{S\times D}$ contains one bidirectionally encoded representation for each EEG window. Two classification heads are attached to $H_{i}$. The primary head applies the same classifier to every contextualized token and produces a two-class prediction for each window. The classifier consists of layer normalization, a 64-dimensional hidden linear layer, GELU activation, dropout and a two-unit output layer.

For the s-th window, the predicted class probabilities are given by

(18) $$p_{i,s}^{win}=\mathrm{softmax}\left(C_{win}\left(h_{i,s}\right)\right),\;\;p_{i,s}^{win}\in R^{2},$$

The two elements correspond to the probabilities of the healthy-control and depression classes, respectively. The class with the higher probability is taken as the prediction for the current EEG window.

The auxiliary head first assigns an attention score to each contextualized window token and calculates their weighted sum. This sequence representation is processed by a second two-class classifier to produce one prediction for the complete S-window segment. The auxiliary output is used only to regularize sequence representation learning; the window-level output remains the primary prediction used for model evaluation.

The model is optimized using a combination of the window-level classification loss and the auxiliary sequence-level loss:

(19) $$L=L_{win}+\lambda_{seq}L_{seq},$$

where both terms are calculated using cross-entropy loss and $\lambda_{seq}$ controls the contribution of sequence-level supervision. In the current implementation, $\lambda_{seq}=0.5$. During evaluation, only the window-level predictions are used as the primary outputs, whereas the sequence-level head acts as an auxiliary training constraint.

## 3. Experiments and Results

### 3.1. Datasets

#### 3.1.1. Dataset Introduction

To evaluate FSC-BiMamba under different EEG acquisition conditions, experiments were conducted on two publicly available resting-state EEG datasets: the MODMA dataset [34] and the Mumtaz2016 dataset [35]. Both datasets provide binary diagnostic labels for major depressive disorder (MDD) and healthy controls (HCs), but differ in recording equipment, data format, channel configuration and acquisition protocol. The three-channel resting-state EEG subset of MODMA was used as the primary evaluation dataset, whereas Mumtaz2016 served as a supplementary validation dataset to assess the applicability of the proposed model under a different acquisition setting. The main characteristics of the two datasets are summarized in Table 1.

The MODMA dataset, established at the Second Hospital of Lanzhou University, includes a three-channel resting-state EEG subset of 55 participants, comprising 26 patients with MDD and 29 HCs. All patients with MDD were diagnosed according to the criteria specified in the fourth edition of the Diagnostic and Statistical Manual of Mental Disorders (DSM-IV). Resting-state EEG signals were recorded from the Fp1, Fpz and Fp2 channels at a sampling rate of 250 Hz. For the present analysis, the first 500 s of each eligible recording were retained. After excluding recordings with insufficient duration or an inadequate number of clean windows following artefact rejection, the final experimental cohort comprised 54 participants, including 26 patients with MDD and 28 HCs.

The Mumtaz2016 dataset contains EEG recordings from 64 participants, including 34 patients with MDD and 30 HCs. Participants were recruited from the outpatient clinics of Hospital Universiti Sains Malaysia (HUSM), and MDD diagnoses were established by qualified clinicians according to the DSM-IV criteria. EEG signals were acquired using the Brain Master Discovery system with a 19-channel electrode configuration at a sampling rate of 256 Hz. Each participant underwent a 5 min eyes-closed (EC) recording and a 5 min eyes-open (EO) recording under resting-state conditions. Only the EC recordings were used in this study, and the Fp1, Fz and Fp2 channels were selected to match the three-channel frontal input configuration of the proposed model. After participants with incomplete EEG recordings were excluded, the final experimental cohort comprised 58 participants, including 30 patients with MDD and 28 HCs.

#### 3.1.2. Data Preprocessing

Before applying the common preprocessing pipeline, dataset-specific record selection and signal harmonization were performed. All recordings were organized into a three-channel frontal EEG input format and were temporally truncated or resampled according to the acquisition settings of each dataset. For MODMA, the raw text EEG files were matched to their corresponding diagnostic labels using the subject metadata table, and the first 500 s of each retained recording was used for analysis. For Mumtaz2016, the diagnostic label and recording condition were identified from the EDF filename, and only EC resting-state recordings were retained. The Fp1, Fz and Fp2 channels were selected. Because the original sampling rate was 256 Hz, the signals were resampled to 250 Hz using polyphase resampling. In contrast to MODMA, the complete usable duration of each Mumtaz2016 EC recording was included.

**Figure 2:**
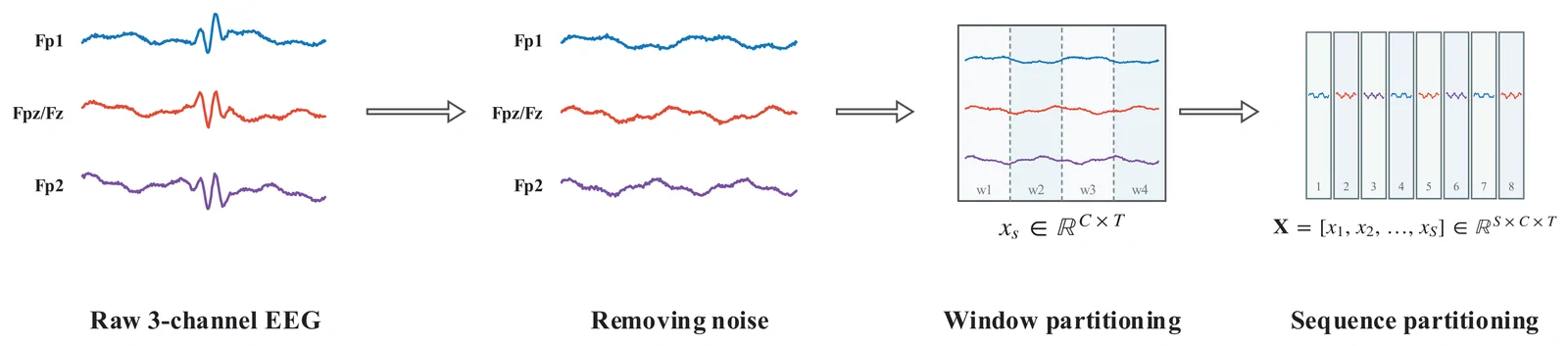
Schematic illustration of the three-channel EEG preprocessing pipeline.

The EEG preprocessing pipeline is illustrated in Figure 2. For both datasets, the signals were band-pass-filtered between 0.5 and 45 Hz using a fourth-order Butterworth filter. Forward and reverse filtering was applied to obtain a zero-phase response. The filtered signals were subsequently segmented into non-overlapping 2 s windows, with each window containing three channels and 500 samples per channel. Artefact rejection was performed using both maximum absolute amplitude and root-mean-square (RMS) amplitude criteria. However, because the maximum-amplitude and RMS criteria are primarily sensitive to prominent amplitude abnormalities, subtle myogenic activity may remain in some retained windows and potentially influence high-frequency β-band and γ-band features. Finally, z-score normalization was applied to the valid EEG windows to standardize signal amplitudes and ensure a consistent input scale for model training. After preprocessing, each retained sample corresponded to a 2 s EEG window represented as $x_{s}\in R^{C\times T}$. To construct the input sequences for cross-window temporal modelling, eight temporally consecutive non-overlapping windows from the same participant were grouped into a 16 s window sequence, with a stride of eight windows. Only eight temporally consecutive clean windows from the same participant were grouped into a sequence. Candidate sequences containing or spanning an artefact-rejected window were excluded from the analysis.

### 3.2. Experimental Settings

#### 3.2.1. Implementation Details

FSC-BiMamba was implemented in Python 3.10.20 using PyTorch 2.11.0 and CUDA 12.8. The experiments were executed on an NVIDIA GeForce RTX 3060 GPU. The model was trained for a maximum of 120 epochs with a batch size of 32. AdamW was used with an initial learning rate of $3\times 10^{-4}$ and a weight decay of $1\times 10^{-3}$. The learning rate was adjusted using cosine annealing. Class-weighted cross-entropy with label smoothing of 0.05 was used to alleviate class imbalance. The total objective combined the window-level classification loss with a sequence-level auxiliary loss weighted by 0.5. Gradient clipping was applied with a maximum norm of 1.0. Early stopping was activated after a minimum of 20 epochs if validation-balanced accuracy did not improve for 25 consecutive epochs.

#### 3.2.2. Verification Strategy

For both datasets, FSC-BiMamba was evaluated using stratified, subject-independent fivefold cross-validation with window-level supervision and evaluation. Participants were stratified by diagnostic label and assigned to five mutually exclusive folds. In each iteration, one fold was used for testing, the next fold in cyclic order for validation, and the remaining three folds for training. All windows and sequences from each participant remained in the same partition, preventing overlap across the training, validation, and test sets.

Temporally consecutive clean 2 s windows from each participant were grouped into non-overlapping sequences of eight. Sequences did not span rejected intervals or recording-session boundaries. The sequences provided cross-window temporal context, while each window retained its diagnostic label and yielded an individual prediction. Training loss, validation balanced accuracy, and test metrics were calculated from window-level outputs without participant-level aggregation. The checkpoint with the highest validation balanced accuracy was evaluated on the corresponding test fold. Test data were excluded from model training, model selection, and threshold calibration. Final metrics were reported as the mean and standard deviation across the five test folds.

#### 3.2.3. Evaluation Metrics

The classification performance of FSC-BiMamba was evaluated using four metrics: accuracy, precision, recall and F1-score. Major depressive disorder (MDD) was designated as the positive class and healthy control (HC) as the negative class. For each test fold, the predicted probabilities were converted into binary labels using a fixed threshold of 0.5.

Accuracy represents the proportion of correctly classified windows among all test windows and is defined as

(20) $$Accuracy=\frac{TP+TN}{TP+TN+FP+FN},$$

where TP and TN denote the numbers of MDD and HC windows correctly classified, respectively. FP denotes the number of HC windows incorrectly classified as MDD, whereas FN denotes the number of MDD windows incorrectly classified as HC.

Precision measures the proportion of true MDD windows among all windows classified as MDD:

(21) $$Precision=\frac{TP}{TP+FP}.$$

Recall, also referred to as sensitivity, quantifies the proportion of MDD windows correctly identified by the model:

(22) $$Recall=\frac{TP}{TP+FN}.$$

The F1-score is the harmonic mean of precision and recall and provides a joint assessment of these two measures:

(23) $$F1\text{-}score=2\times \frac{Precision\times Recall}{Precision+Recall}.$$

All metrics were calculated separately for the held-out test set in each fold and are reported as the mean and standard deviation across the five folds. Confusion matrices were also constructed from the test predictions to characterize class-specific performance. The rows and columns of each confusion matrix represent the ground-truth and predicted labels, respectively, allowing false-negative MDD predictions and false-positive HC predictions to be examined separately.

### 3.3. Depression Detection Performance

#### 3.3.1. Results on the MODMA Dataset

Table 2 reports the fold-wise window-level classification performance of FSC-BiMamba on the MODMA dataset under strict subject-independent five-fold cross-validation. FSC-BiMamba achieved an average accuracy of 82.70±2.37%, precision of 82.50±1.65%, recall of 81.36±5.33% and F1-score of 81.86±2.90% on the MODMA dataset. The relatively small standard deviations indicate that the learned representations remained stable across different groups of held-out subjects. This consistency may be partly attributed to the standardized acquisition protocol and fixed three-channel configuration of MODMA, which reduce non-clinical variability in the model input.

Across the five folds, Fold 3 achieved the best overall performance, with an accuracy of 86.69% and an F1-score of 86.49%. Its simultaneously high precision of 84.60% and recall of 88.46% suggest that the EEG characteristics of the held-out participants were well represented by the patterns learned from the training subjects, resulting in improved separation between MDD and HC windows. In contrast, Fold 2 yielded the lowest accuracy of 80.63%, primarily because its recall decreased to 76.44% while precision remained at 82.10%. This result indicates that the performance reduction was mainly caused by missed MDD windows, possibly because some MDD participants in this fold exhibited less pronounced or more heterogeneous depression-related EEG patterns. The remaining folds achieved accuracies between 81.54% and 82.83%, confirming that the model maintained relatively stable window-level performance despite moderate variation in MDD sensitivity.

**Figure 3:**
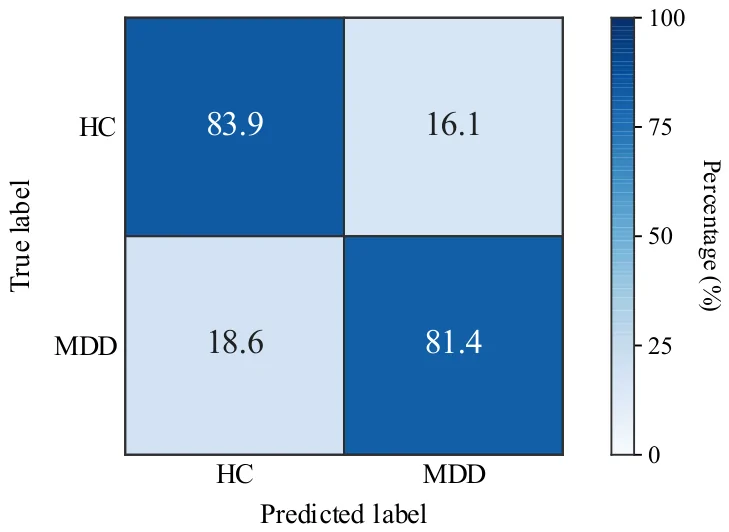
Fold-averaged row-normalized subject-independent window-level confusion matrix of FSC-BiMamba on the MODMA dataset under strict subject-independent five-fold cross-validation. Rows denote ground-truth labels and columns denote predicted labels.

Figure 3 further illustrates the class-specific performance of FSC-BiMamba. The model correctly classified 83.94% of HC windows and 81.36% of MDD windows, and the difference between the two class-specific recognition rates was only 2.58%, indicating relatively balanced discrimination without a pronounced prediction bias towards either diagnostic group. The corresponding false-positive and false-negative rates were 16.06% and 18.64%, respectively. The slightly higher false-negative rate may partly arise from the use of participant-level diagnostic labels for individual 2 s windows, as not every window from an MDD participant necessarily contains an equally pronounced depression-related pattern. Consequently, some short MDD windows may resemble normal resting-state EEG activity and be classified as HC. In addition, the limited spatial coverage of the three frontal channels may restrict the detection of subtle or spatially distributed abnormalities, while the fixed decision threshold may assign ambiguous MDD windows with borderline probabilities to the HC class.

#### 3.3.2. Results on the Mumtaz2016 Dataset

Table 3 presents the window-level classification performance of FSC-BiMamba on the Mumtaz2016 dataset under subject-independent five-fold cross-validation. The model achieved an average accuracy of 84.55±8.01%, precision of 89.02±6.13%, recall of 79.73±21.98% and F1-score of 82.14±13.92%. Although the mean accuracy was relatively high, the larger standard deviations of recall and F1-score indicate that MDD sensitivity varied substantially across the held-out subject groups. This variation may reflect greater inter-subject heterogeneity in the MDD recordings and the stronger influence of individual participants when each test fold contains a limited number of subjects.

Across the five folds, Fold 2 achieved the best overall performance, with an accuracy of 93.69% and an F1-score of 93.20%. Its closely matched precision of 93.51% and recall of 92.89% indicate balanced classification, possibly because the EEG characteristics of the held-out subjects were consistent with the MDD and HC patterns represented in the training data. By contrast, Fold 3 produced the lowest accuracy of 72.10% and F1-score of 58.21%. Although its precision remained high at 95.63%, recall declined sharply to 41.84%, indicating a conservative decision pattern in which many MDD windows were assigned to the HC class. This behaviour may reflect a distributional difference between the MDD participants in Fold 3 and those used for training. The remaining folds achieved accuracies between 82.54% and 87.43%, but their different precision–recall profiles further indicate that the principal source of fold-to-fold variation was the model’s sensitivity to MDD patterns.

**Figure 4:**
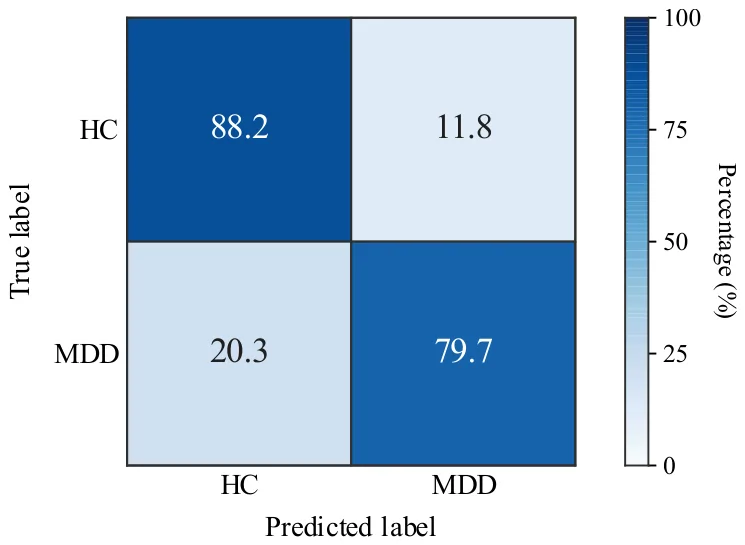
Fold-averaged row-normalized subject-independent window-level confusion matrix of FSC-BiMamba on the Mumtaz2016 dataset under strict subject-independent five-fold cross-validation. Rows denote ground-truth labels and columns denote predicted labels.

A more asymmetric error distribution is evident in Figure 4. The model correctly classified 88.2% of HC windows but 79.7% of MDD windows, yielding false-positive and false-negative rates of 11.8% and 20.3%, respectively. The higher false-negative rate is consistent with the comparatively lower and more variable recall reported in Table 3, particularly the low MDD sensitivity observed in Fold 3. This asymmetry may be related to greater inter-subject heterogeneity among MDD participants, causing some unseen MDD windows to lie closer to the HC distribution in the learned feature space and consequently fall below the fixed decision threshold.

Overall, FSC-BiMamba achieved comparable mean window-level classification performance on the two datasets but exhibited different stability profiles. The smaller fold-to-fold variation on MODMA may be associated with its standardized three-channel acquisition and more consistent input configuration. By contrast, the greater variation observed on Mumtaz2016 may reflect differences in cohort composition, recording conditions and frontal electrode configuration. These results provide complementary evidence for the cross-subject generalization of FSC-BiMamba while highlighting the remaining challenge of reliably identifying heterogeneous MDD patterns in previously unseen participants.

### 3.4. Comparison Experiment

To evaluate the effectiveness and computational efficiency of FSC-BiMamba, we compared it with seven representative machine-learning and deep-learning baselines.

1. SVM maps the input features into a nonlinear feature space through a radial basis function kernel and constructs a maximum-margin decision boundary.
2. EEGNet [10] is a compact EEG-specific convolutional network that combines temporal convolution, depthwise spatial convolution, and separable convolution. These operations capture temporal patterns and inter-channel relationships while maintaining a small parameter count.
3. DeprNet [36] is a deep convolutional framework developed specifically for EEG-based depression detection. It employs stacked convolutional operations to learn discriminative representations directly from EEG signals.
4. EEG Conformer [12] integrates a convolutional front end with Transformer encoders. The convolutional module extracts local EEG patterns, whereas the self-attention mechanism captures long-range dependencies, enabling local and global information to be modelled jointly.
5. 1D-CNN-GRU-ATTN [37] combines one-dimensional convolution, gated recurrent units, and an attention mechanism. The convolutional component extracts local patterns, the recurrent component models sequential dependencies, and the attention mechanism emphasizes informative representations for classification.
6. Improved BiLSTM [38] models sequential dependencies in both temporal directions. Dynamic routing is subsequently used to aggregate informative representations for classification.
7. SSAFNet [39] integrates cross-frequency and spatiotemporal feature extraction with adaptive feature fusion. This design enables complementary spectral and spatiotemporal EEG information to be represented jointly for depression classification.

To ensure a fair comparison, we retained the core architecture of each baseline and adapted only input-dependent components to the common three-channel, 2 s EEG setting. Architecture-specific learning rates were predefined, whereas all neural models shared the same optimizer, training budget, early-stopping rule, and validation-based checkpoint-selection criterion. All settings were fixed before testing, without using test data for hyperparameter selection. Table 4 and Table 5 report subject-independent window-level and subject-level performance under identical subject-independent five-fold splits (mean ± standard deviation). Window-level metrics were computed from individual window predictions, whereas subject-level predictions were obtained by majority voting across all windows from each held-out participant. Statistical significance was assessed using subject-wise paired permutation tests between FSC-BiMamba and each competing model, separately for each dataset, evaluation level, and metric. Within each endpoint, p-values were adjusted across model comparisons using Holm’s procedure; $p_{Holm}<0.05$ was considered significant and is denoted by an asterisk in Table 4 and Table 5.

#### 3.4.1. Comparison Results on the MODMA Dataset

As shown in Table 4, FSC-BiMamba achieved the highest window-level accuracy, recall, and F1-score on the MODMA dataset, reaching 82.70 ± 2.37%, 81.36 ± 5.33%, and 81.86 ± 2.90%, respectively. Compared with SSAFNet, FSC-BiMamba improved accuracy, precision, recall, and F1-score by 3.52, 3.56, 3.92, and 3.69 percentage points, respectively. Although SVM achieved a higher precision, its substantially lower recall resulted in a lower F1-score, indicating a tendency to identify depressive cases more conservatively. In contrast, the relatively balanced precision and recall of FSC-BiMamba indicate that its performance improvement was not achieved by favouring either class.

At the subject level, FSC-BiMamba achieved the highest mean performance across all four metrics, with an accuracy of 79.33 ± 2.30%, a precision of 78.39 ± 6.65%, a recall of 80.10 ± 9.68%, and an F1-score of 78.66 ± 3.27%. Relative to SSAFNet, these results represented improvements of 4.09, 4.21, 2.85, and 4.34 percentage points, respectively. Although the subject-level values were slightly lower than the corresponding window-level results, FSC-BiMamba retained its leading performance when each participant contributed only one final prediction. This reduction may indicate that classification errors were concentrated in a small subset of difficult participants and therefore could not be corrected through majority voting across their windows.

#### 3.4.2. Comparison Results on the Mumtaz2016 Dataset

As presented in Table 5, FSC-BiMamba obtained the highest window-level accuracy and precision on the Mumtaz2016 dataset, reaching 84.55 ± 8.01% and 89.02 ± 6.13%, respectively. Compared with SSAFNet, its accuracy and precision increased by 1.33 and 4.79 percentage points, respectively. Its window-level recall and F1-score were 79.73 ± 21.98% and 82.14 ± 13.92%. The relatively large fold-wise variation, particularly in recall, suggests that window-level performance was sensitive to the composition of the held-out participants, consistent with the limited cohort size of this dataset.

At the subject level, FSC-BiMamba achieved the highest mean values for all four metrics, with an accuracy of 87.88 ± 4.88%, a precision of 90.48 ± 8.75%, a recall of 93.33 ± 7.45%, and an F1-score of 91.88 ± 4.44%. Compared with the best-performing competing model for each metric, FSC-BiMamba improved accuracy, precision, recall, and F1-score by 0.08, 3.81, 3.33, and 4.30 percentage points, respectively. Its subject-level recall and F1-score were also 13.60 and 9.74 percentage points higher than the corresponding window-level results, with considerably lower fold-wise variability. This improvement may be attributed to majority voting, which reduces the influence of isolated erroneous window-level predictions when the predictions across most windows from the same participant are consistent.

Overall, FSC-BiMamba demonstrated competitive performance at both evaluation levels. In particular, its advantage was retained when the evaluation was based on one final prediction per participant, indicating that the observed performance was not confined to window-level assessment. The consistent results are also in line with the intended design of FSC-BiMamba, which integrates local time–frequency representations, explicit frequency-domain descriptors, and cross-window temporal dependencies to characterize sparse frontal EEG signals.

#### 3.4.3. Model Complexity Comparison

To assess parameter efficiency under the same frontal three-channel input setting, Table 6 compares the trainable parameter counts and FP32 parameter storage of the deep learning models after adaptation to the same input configuration and binary classification task. EEGNet is the smallest model, with 2258 trainable parameters and a storage requirement of 0.009 MiB. However, its substantially lower classification performance in Table 4 and Table 5 indicates that the smallest parameter count did not translate into the best performance under the present task setting. FSC-BiMamba contains 93,807 trainable parameters and requires 0.358 MiB of storage. Although it does not have the lowest absolute parameter count, FSC-BiMamba uses 22.68% and 68.03% fewer parameters than EEG Conformer and BiLSTM with dynamic routing, respectively. More substantial reductions of 83.13%, 83.27%, and 99.12% are observed relative to DeprNet, 1D-CNN-GRU-ATTN, and SSAFNet, respectively. This indicates that FSC-BiMamba remained compact relative to most temporal or attention-based deep baselines while achieving higher accuracy on both datasets. Together with its cross-subject window-level performance, these results indicate that FSC-BiMamba provides a favourable trade-off between predictive performance and model size while supporting cross-window temporal modelling.

### 3.5. Ablation Experiments

To assess the contribution of each component in FSC-BiMamba, ablation experiments were conducted by removing TFME, FSDE, ADTF and BiMamba, respectively. All model variants were evaluated using the same preprocessing pipeline, subject-independent five-fold cross-validation strategy and window-level evaluation protocol as the full model. The results on the MODMA and Mumtaz2016 datasets are reported in Table 7 and Table 8, respectively.

On the MODMA dataset, removing TFME caused the largest performance drop, reducing accuracy from 82.70% to 73.52% and F1-score from 81.86% to 70.87%. This indicates that time–frequency encoding is central to extracting discriminative oscillatory patterns from short frontal EEG windows. Removing FSDE or BiMamba also led to clear decreases in accuracy and F1-score, suggesting that explicit statistical descriptors and cross-window temporal modelling provide additional complementary information. The smaller decrease caused by removing ADTF indicates that the two branches already contain useful representations, while adaptive token fusion further improves their integration. On the Mumtaz2016 dataset, removing BiMamba produced the largest performance decrease, with accuracy falling from 84.55% to 77.02%. This suggests that temporal context is particularly important on this dataset, possibly due to stronger inter-subject variability under the same frontal three-channel setting. Removing TFME, FSDE or ADTF resulted in smaller but consistent decreases in overall performance. Notably, the w/o TFME variant showed slightly higher recall but lower precision, indicating a shift toward more positive predictions rather than a genuine improvement. These results support the design of FSC-BiMamba, in which time–frequency encoding, statistical descriptor encoding, adaptive fusion and bidirectional temporal modelling jointly improve frontal sparse-channel EEG depression detection.

## 4. Discussion

### 4.1. Sensitivity Analysis of Window and Sequence Configuration

The sensitivity analysis presented in Table 9 provides empirical support for using 2 s windows and eight windows per sequence. With the sequence length fixed at eight windows, the 2 s setting achieved the highest mean accuracy and F1 score on both datasets. Compared with the 1 s setting, it increased accuracy by 7.06 percentage points on MODMA and 2.19 percentage points on Mumtaz2016. Increasing the window duration to 4 s also reduced both metrics relative to the 2 s setting. These results suggest that 2 s windows may provide an appropriate balance between temporal resolution and reliable feature estimation. Specifically, 1 s windows may contain insufficient temporal information for robust spectral characterization, whereas 4 s windows may be less sensitive to transient EEG variations. This trade-off may benefit both the short-term time–frequency representations learned by TFME and the frequency-domain descriptors encoded by FSDE.

With the window duration fixed at 2 s, sequences of eight windows again achieved the highest mean accuracy and F1 score on both datasets. However, the difference between four and eight windows was small on Mumtaz2016, suggesting that the benefit of additional temporal context may depend on dataset characteristics. Increasing the sequence length to 16 windows did not improve performance. This result may reflect increased redundancy or non-stationarity in longer EEG sequences. Overall, the 2 s, eight-window configuration was the most consistently effective among the evaluated settings and provided BiMamba with a 16 s temporal context for cross-window modelling.

From a computational perspective, each directional Mamba scan has linear time complexity with respect to the number of window tokens when the hidden and state dimensions are fixed. Bidirectional processing increases the constant computational factor but preserves this linear scaling. Inference activation memory also grows approximately linearly with sequence length, whereas the 0.358 MiB parameter storage reported in Table 6 remains independent of sequence length. Consequently, the selected eight-window configuration limits the additional computational and activation-memory costs associated with longer sequences while retaining the best overall performance.

### 4.2. ROC and Precision–Recall Analysis

**Figure 5:**
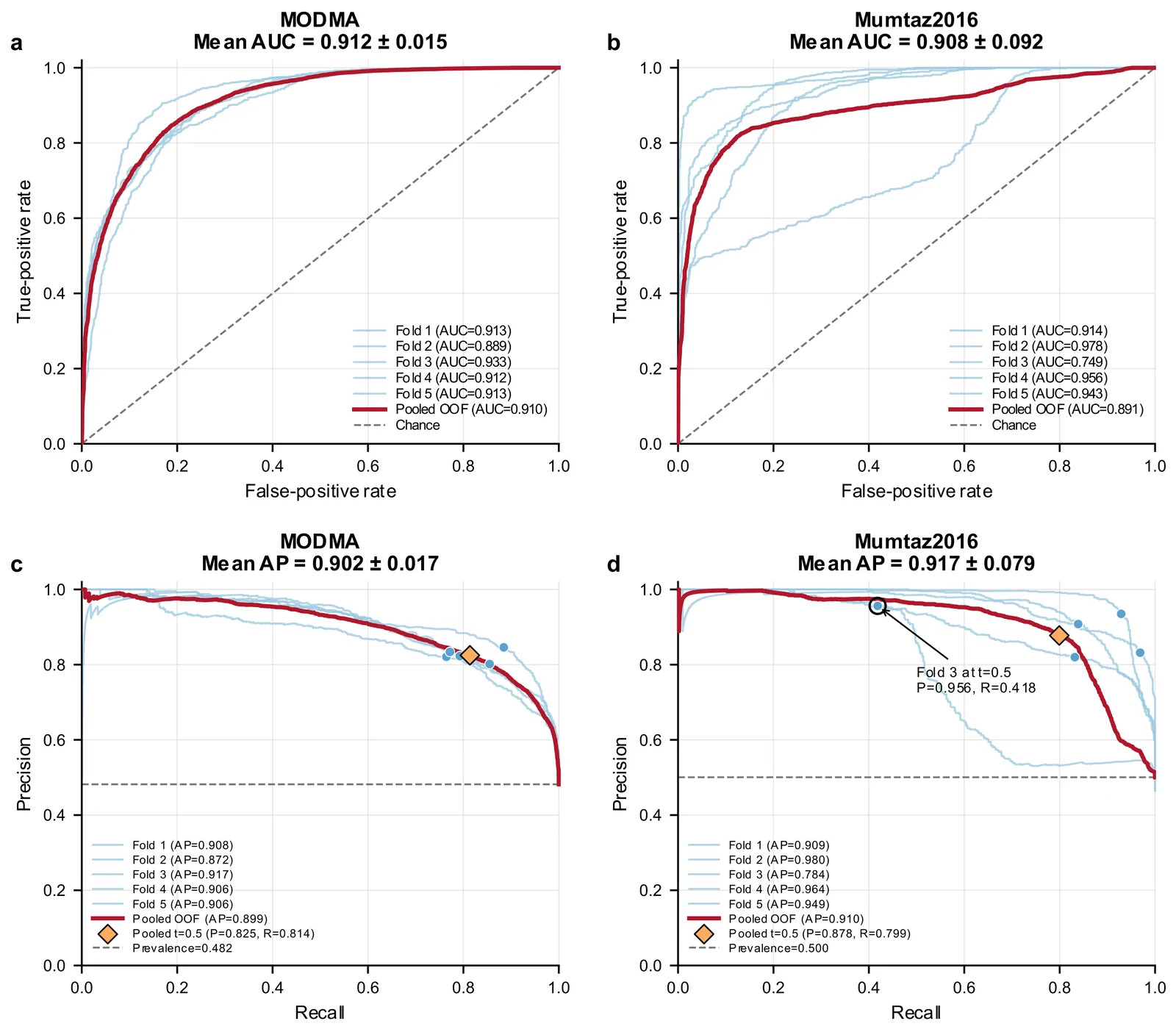
Fold-wise and pooled out-of-fold receiver operating characteristic and precision–recall curves of FSC-BiMamba. (**a**,**b**) ROC curves on MODMA and Mumtaz2016, respectively. (**c**,**d**) PR curves on MODMA and Mumtaz2016.

To provide a threshold-independent evaluation of FSC-BiMamba, fold-wise and pooled out-of-fold (OOF) ROC and precision–recall curves were examined (Figure 5). On MODMA, the model achieved a mean AUC of 0.912 ± 0.015 and a mean AP of 0.902 ± 0.017, indicating stable discrimination across folds. On Mumtaz2016, the corresponding values were 0.908 ± 0.092 and 0.917 ± 0.079, although greater fold-wise variability was observed. In particular, Fold 3 yielded an AUC of 0.749 and an AP of 0.784. At the threshold of 0.5, this fold achieved high precision (0.956) but low recall (0.418), reflecting a conservative operating point. Its lower AUC and AP may partly reflect variability in the characteristics of the held-out participants.

At the same threshold, the pooled OOF predictions yielded precision and recall values of 0.825 and 0.814 on MODMA, and 0.878 and 0.799 on Mumtaz2016, respectively. These results demonstrate strong dataset-level discrimination while confirming that the precision–recall balance varies with the operating threshold and test-fold composition. Therefore, 0.5 was retained as a consistent reference threshold for model comparison rather than regarded as universally optimal. In future applications, the operating threshold should be selected on an independent validation set according to the intended clinical objective.

### 4.3. Joint Channel–Frequency Feature Attribution

**Figure 6:**
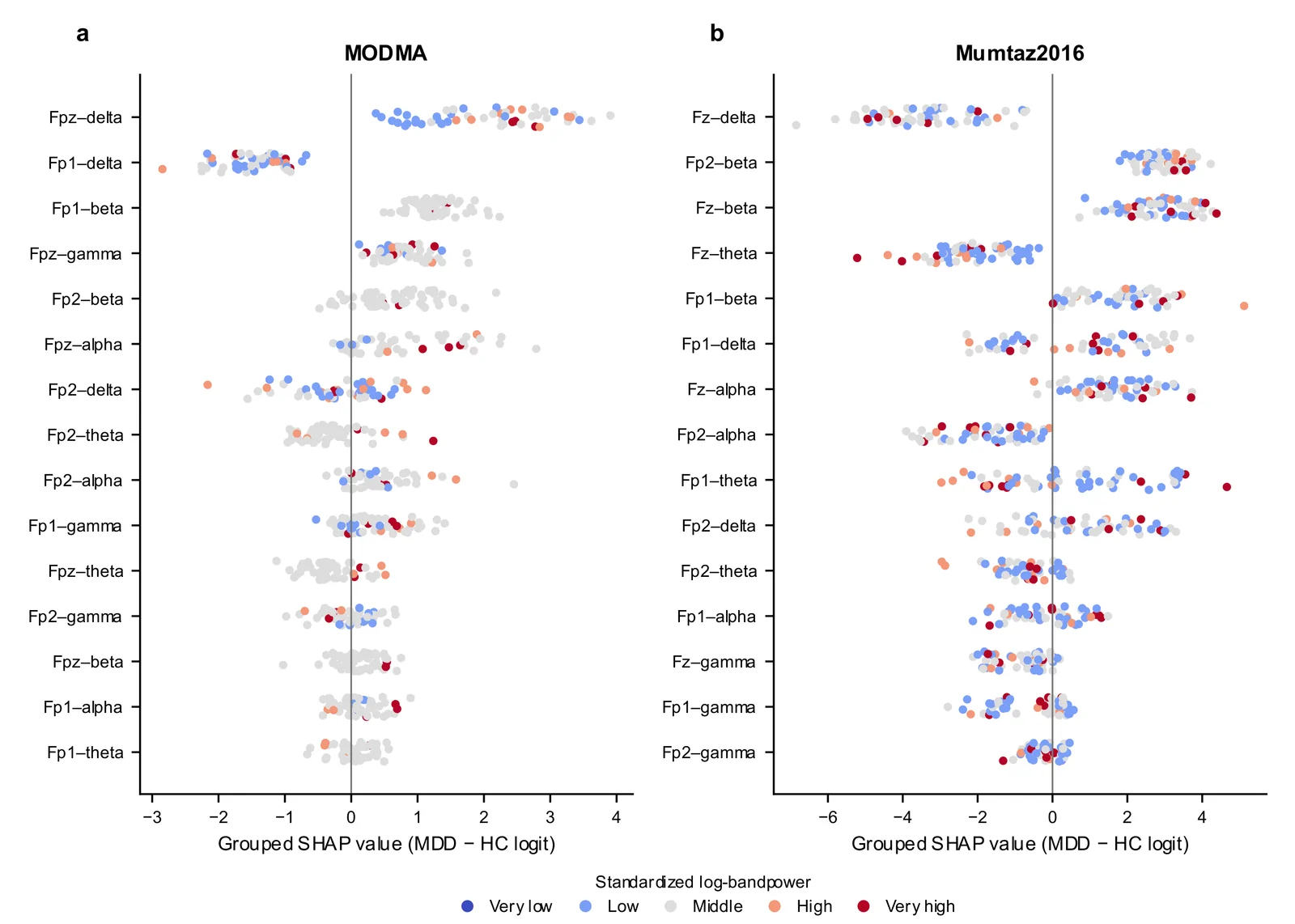
Interpretable attribution of channel–frequency features using grouped SHAP. Participant-level SHAP distributions for the 15 channel–frequency combinations in (**a**) MODMA and (**b**) Mumtaz2016. Features are ranked according to their mean absolute SHAP values.

Grouped SHAP analysis further opened the input-level decision process by quantifying both the magnitude and direction of each channel–frequency contribution (Figure 6). Fpz–delta, Fp1–delta, and Fp1–beta were the most influential features in MODMA, whereas Fz–delta, Fp2–beta, and Fz–beta were dominant in Mumtaz2016. Positive and negative SHAP values indicate whether a feature shifts the model output toward MDD or HC, respectively. Thus, the analysis identifies not only which features the model considers important but also how they contribute to individual predictions.

### 4.4. Frequency-Band and Channel-Specific Contributions

**Figure 7:**
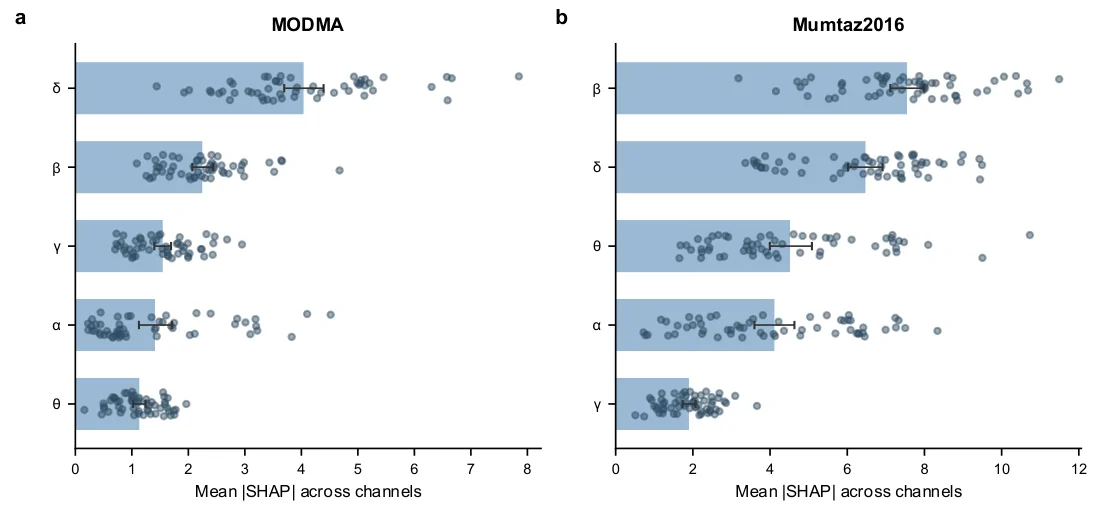
Frequency-band contributions to FSC-BiMamba predictions. Participant-level mean absolute SHAP values aggregated across EEG channels for the delta, theta, alpha, beta, and gamma bands in (**a**) MODMA and (**b**) Mumtaz2016.

**Figure 8:**
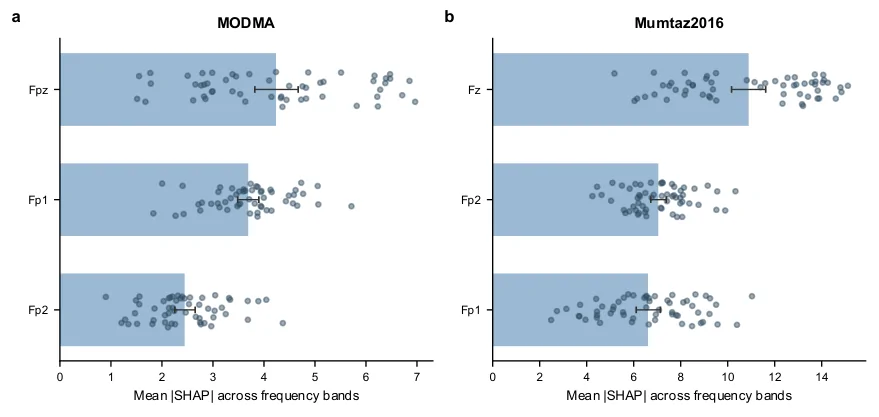
Contributions of individual frontal EEG channels. Participant-level mean absolute SHAP values aggregated across the five frequency bands for each EEG channel in (**a**) MODMA and (**b**) Mumtaz2016.

Aggregated SHAP values further clarified the spectral and spatial contributions of the model. Delta activity showed the largest contribution in MODMA, whereas beta and delta activity predominated in Mumtaz2016 (Figure 7). At the channel level, the midline frontal channel contributed most strongly in both datasets—Fpz in MODMA and Fz in Mumtaz2016—while the bilateral frontal channels provided complementary information (Figure 8). These findings support the relevance of frontal spectral patterns to the model predictions.

### 4.5. Visualization of Adaptive Fusion Behaviour in the ADTF Module

**Figure 9:**
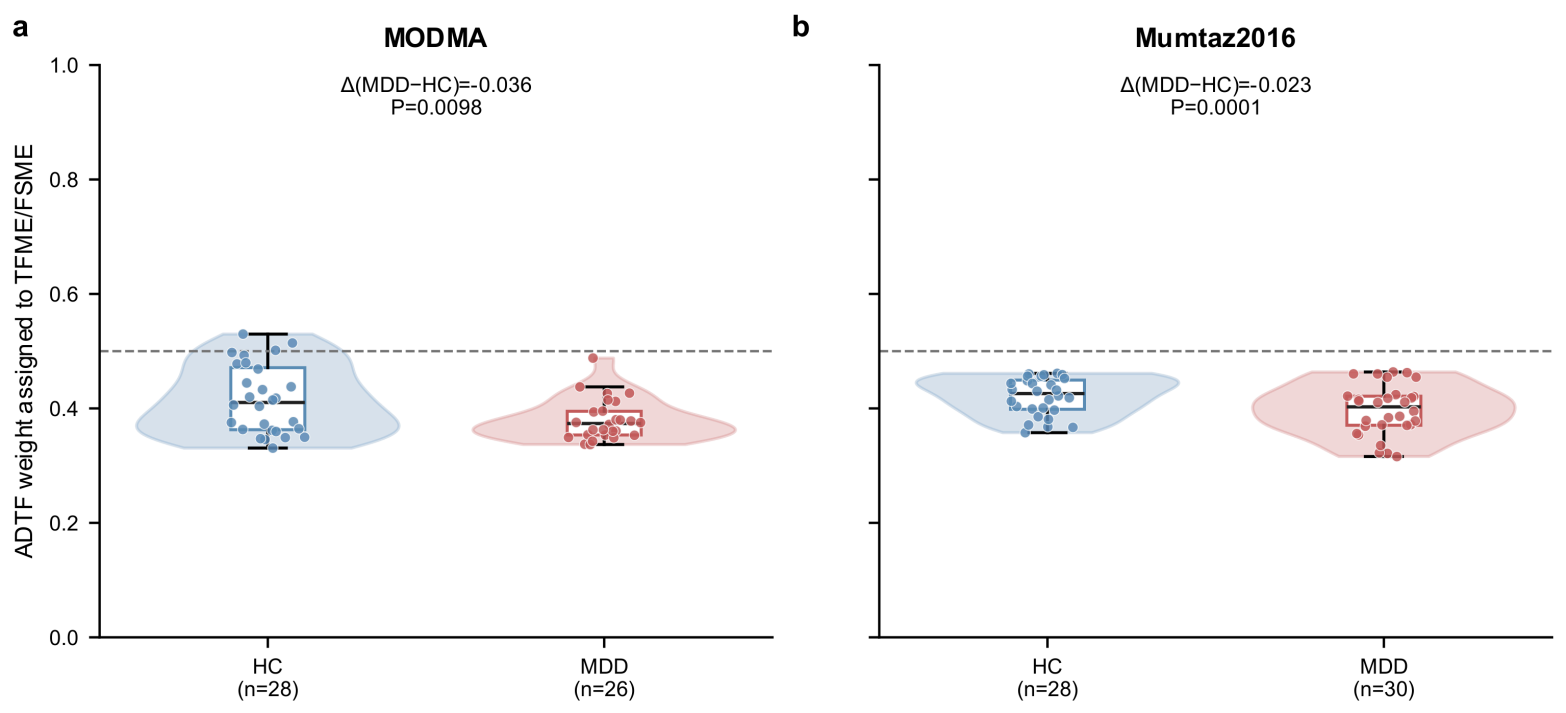
Interpretable visualization of the adaptive feature-fusion behaviour of ADTF. Distributions of the learned allocation to the TFME pathway in the HC and MDD groups for (**a**) MODMA and (**b**) Mumtaz2016. The complementary allocation to the FSDE pathway is defined as one minus the TFME gate value.

Figure 9 provides an interpretable view of the internal fusion mechanism of the ADTF module. The mean TFME gate values were 0.398 in MODMA and 0.408 in Mumtaz2016, indicating that FSC-BiMamba generally assigned greater weight to the complementary FSDE pathway while retaining information from both representations. The TFME allocation was also lower in the MDD group than in the HC group in both datasets (MODMA: Δ = −0.036, *p* = 0.0098; Mumtaz2016: Δ = −0.023, *p* < 0.001). The violin and box plots further show inter-participant variability and substantial overlap between the clinical groups. These findings show that ADTF does not apply a fixed fusion rule but adaptively adjusts the balance between time–frequency and statistical representations according to the input.

### 4.6. Neurophysiological Interpretation and Clinical Implications

The attribution and gating results clarify the EEG information used by FSC-BiMamba. Delta activity contributed most strongly in MODMA, whereas both delta and beta were prominent in Mumtaz2016, indicating that the model integrates information from different frequency bands rather than relying on a single spectral marker. The predominance of Fpz or Fz, together with complementary contributions from the bilateral frontal channels, suggests that the model combines midline frontal activity with lateral information. This bilateral information may support the asymmetry measures encoded by FSDE. The greater average ADTF allocation to FSDE further indicates that its explicit descriptors collectively contributed substantially to prediction. Within FSDE, covariance features characterize amplitude co-fluctuations across frontal channels, whereas phase-based connectivity measures estimate their frequency-specific statistical coupling. TFME additionally captures local time–frequency patterns, while BiMamba models the evolution of these representations across consecutive windows.

From a clinical perspective, FSC-BiMamba is intended to support lightweight preliminary screening for depression rather than stand-alone diagnosis. Its three-channel frontal configuration reduces the acquisition burden associated with dense-channel EEG systems and may facilitate future use in portable or resource-limited settings. Within a potential screening pathway, the model could provide complementary EEG-based risk information to help identify individuals who may benefit from further assessment using standardized questionnaires, structured clinical interviews, and professional psychiatric evaluation. Practical suitability will also depend on model calibration, operating-threshold selection, and the acceptable trade-off between false-negative and false-positive decisions in the intended population. FSC-BiMamba should therefore be regarded as a research-stage screening framework, and prospective validation in larger and more heterogeneous clinical cohorts is required before clinical deployment.

### 4.7. Limitations and Future Research

Despite its suitability for lightweight EEG screening, FSC-BiMamba has several limitations. First, the relatively small cohorts in the two independently collected public datasets limit the generalizability of the findings. Although subject-independent cross-validation reduced the risk of information leakage, it could not compensate for limited participant diversity or uncertainty arising from small held-out cohorts. The fold-to-fold variability observed on Mumtaz2016 also indicates that performance remains sensitive to the composition of the held-out participants. Furthermore, the current group-level SHAP analyses cannot determine whether channel–frequency attribution patterns vary by age or gender. Future studies should therefore evaluate FSC-BiMamba in larger, prospectively recruited multicenter cohorts and perform age- and gender-stratified attribution analyses.

Second, both datasets included only resting-state EEG recordings, limiting conclusions about performance under other acquisition paradigms and real-world screening conditions. External validation should include task-based and ambulatory EEG, different acquisition systems, and alternative sparse-channel configurations. Such validation would provide stronger evidence of model robustness and clinical applicability.

## 5. Conclusions

This study proposed FSC-BiMamba for depression detection from frontal sparse-channel EEG, aiming to address both the limited spatial information of compact EEG recordings and the insufficient modelling of temporal context across consecutive windows. In FSC-BiMamba, the Time–Frequency Map Encoder (TFME) captures local oscillatory patterns from time–frequency maps, while the Frequency-Domain Statistical Descriptor Encoder (FSDE) preserves explicit spectral, asymmetry, connectivity and covariance descriptors. The Adaptive Dual-Token Fusion (ADTF) module integrates these complementary representations, and the bidirectional Mamba (BiMamba) module further models cross-window temporal dependencies. Among the compared models, FSC-BiMamba achieved the highest accuracy on both datasets and provided a favourable trade-off between classification performance and model size, demonstrating the effectiveness of the proposed design for subject-independent depression recognition. Future studies will assess generalisability using larger, heterogeneous EEG datasets, and extend FSC-BiMamba to multimodal physiological data with interpretable modelling strategies to improve robustness and transparency.

## Figures and Tables

**Table 1:** Detailed comparison of the two datasets.

Factor	MODMA	Mumtaz2016
Original Subjects	55	64
MDD/HC	26/29	34/30
Culture	China	Malaysia
Recording condition	Resting-state EEG	EC/EO resting-state EEG
Sampling rate	250 Hz	256 Hz
Number of channels	3	19
EEG equipment	A wearable three-electrode EEG collector	The Brain Master Discovery system

**Table 2:** Fold-wise subject-independent window-level classification performance of FSC-BiMamba on the MODMA dataset under strict subject-independent five-fold cross-validation.

Fold	Accuracy (%)	Precision (%)	Recall (%)	F1-Score (%)
Fold 1	82.83	80.17	85.50	82.75
Fold 2	80.63	82.10	76.44	79.17
Fold 3	86.69	84.60	88.46	86.49
Fold 4	81.79	82.28	79.25	80.73
Fold 5	81.54	83.38	77.16	80.15
Mean ± SD	82.70 ± 2.37	82.50 ± 1.65	81.36 ± 5.33	81.86 ± 2.90

**Table 3:** Fold-wise subject-independent window-level classification performance of FSC-BiMamba on the Mumtaz2016 dataset under strict subject-independent five-fold cross-validation.

Fold	Accuracy (%)	Precision (%)	Recall (%)	F1-Score (%)
Fold 1	82.54	81.99	83.17	82.58
Fold 2	93.69	93.51	92.89	93.20
Fold 3	72.10	95.63	41.84	58.21
Fold 4	87.43	83.18	96.88	89.51
Fold 5	86.97	90.80	83.88	87.20
Mean ± SD	84.55 ± 8.01	89.02 ± 6.13	79.73 ± 21.98	82.14 ± 13.92

**Table 4:** Comparison of subject-independent classification performance on the MODMA dataset.

Verification	Model	Accuracy (%)	Precision (%)	Recall (%)	F1-Score (%)
window-level	SVM	58.85 ± 4.84 *	89.79 ± 9.73 *	47.59 ± 5.11 *	52.73 ± 5.46 *
	EEGNet	62.91 ± 5.24 *	63.82 ± 8.76 *	52.22 ± 7.67 *	62.74 ± 8.93 *
	DeprNet	65.77 ± 2.42 *	70.72 ± 4.35 *	50.00 ± 7.86 *	58.22 ± 4.90 *
	EEG Conformer	66.79 ± 2.72 *	69.67 ± 4.63 *	55.58 ± 4.02 *	61.71 ± 2.97 *
	1D-CNN-GRU-ATTN	72.63 ± 1.02 *	73.29 ± 3.69 *	68.00 ± 3.60 *	70.50 ± 1.88 *
	Improved BiLSTM	76.53 ± 2.64 *	76.15 ± 3.38 *	74.86 ± 3.47 *	75.45 ± 2.68 *
	SSAFNet	79.18 ± 1.31 *	78.94 ± 1.13 *	77.44 ± 2.17 *	78.17 ±1.54 *
	FSC-BiMamba	**82.70 ± 2.37**	82.50 ± 1.65	**81.36 ± 5.33**	**81.86 ± 2.90**
Subject-level	SVM	54.18 ± 14.61 *	52.57 ± 15.95 *	43.33 ± 26.87 *	44.71 ± 22.05 *
	EEGNet	61.64 ± 9.08 *	68.33 ± 20.75 *	54.67 ± 18.50 *	56.56 ± 8.14 *
	DeprNet	66.45 ± 5.98 *	69.67 ± 7.85 *	53.33 ± 12.47 *	59.88 ± 10.08 *
	EEG Conformer	63.12 ± 7.84 *	68.67 ± 18.80 *	58.67 ± 21.81 *	59.17 ± 10.14 *
	1D-CNN-GRU-ATTN	66.67 ± 9.56 *	68.72 ± 9.40 *	65.33 ± 14.91 *	66.20 ± 10.20 *
	Improved BiLSTM	70.06 ± 16.36 *	72.29 ± 19.21 *	71.67 ± 9.23 *	69.57 ± 11.11 *
	SSAFNet	75.24 ± 4.64 *	74.18 ± 6.79 *	77.25 ± 6.32 *	74.32 ± 4.34 *
	FSC-BiMamba	**79.33 ± 2.30**	**78.39 ± 6.65**	**80.10 ± 9.68**	**78.66 ± 3.27**

Bold values indicate the highest mean performance for each metric at each evaluation level. * indicates a statistically significant difference from FSC-BiMamba after Holm correction ($P_{Holm}<0.05$).

**Table 5:** Comparison of subject-independent classification performance on the Mumtaz2016 dataset.

Verification	Model	Accuracy (%)	Precision (%)	Recall (%)	F1-Score (%)
window-level	SVM	72.12 ± 15.65 *	70.41 ± 17.75 *	78.90 ± 20.57 *	73.65 ± 16.72 *
	EEGNet	79.96 ± 6.88 *	79.72 ± 5.78 *	79.97 ± 14.25 *	79.41 ± 8.50 *
	DeprNet	77.24 ± 8.85 *	81.02 ± 12.35 *	75.48 ± 24.06 *	75.10 ± 14.05 *
	EEG Conformer	80.44 ± 10.86 *	79.41 ± 12.71 *	86.91 ± 12.78 *	82.07 ± 8.74 *
	1D-CNN-GRU-ATTN	75.12 ± 5.40 *	78.19 ± 6.30 *	72.60 ± 21.42 *	73.25 ± 10.49 *
	Improved BiLSTM	78.50 ± 3.20 *	78.83 ± 3.38 *	78.82 ± 14.90 *	77.98 ± 6.28 *
	SSAFNet	83.22 ± 4.87 *	84.23 ± 7.38 *	82.37 ± 10.21 *	82.89 ± 5.94 *
	FSC-BiMamba	**84.55 ± 8.01**	**89.02 ± 6.13**	79.73 ±21.98	82.14 ± 13.92
Subject-level	SVM	72.42 ± 20.00 *	71.67 ± 22.85 *	80.00 ± 27.39 *	74.26 ± 21.95 *
	EEGNet	79.24 ± 7.74 *	82.62 ± 16.14 *	83.33 ± 20.41 *	80.04 ± 9.20 *
	DeprNet	83.18 ± 24.23 *	81.81 ± 24.63 *	83.33 ± 28.87 *	82.40 ± 26.64 *
	EEG Conformer	84.55 ± 6.91 *	84.29 ± 10.29 *	90.00 ± 22.36 *	84.54 ± 10.54 *
	1D-CNN-GRU-ATTN	86.21 ± 9.56 *	84.81 ± 9.40 *	90.00 ± 14.91 *	86.82 ± 10.20 *
	Improved BiLSTM	86.06 ± 16.16 *	86.29 ± 19.21 *	86.67 ± 9.13 *	87.57 ± 12.11 *
	SSAFNet	87.80 ± 7.65 *	86.67 ± 7.45 *	83.33 ± 11.79 *	87.58 ± 10.19 *
	FSC-BiMamba	**87.88 ± 4.88**	**90.48 ± 8.75**	**93.33 ± 7.45**	**91.88 ± 4.44**

Bold values indicate the highest mean performance for each metric at each evaluation level. * indicates a statistically significant difference from FSC-BiMamba after Holm correction ($P_{Holm}<0.05$).

**Table 6:** Comparison of trainable parameter counts and FP32 parameter memory across deep learning models adapted to the three-channel input setting.

Model	Trainable Parameters	FP32 Parameter Memory (MiB)
EEGNet	2258	0.009
DeprNet	556,018	2.121
EEG Conformer	121,322	0.463
1D-CNN-GRU-ATTN	560,771	2.139
Improved BiLSTM	293,392	1.119
SSAFNet	10,635,150	40.570
FSC-BiMamba	93,807	0.358

**Table 7:** Ablation results of FSC-BiMamba on the MODMA dataset.

Model	Accuracy (%)	Precision (%)	Recall (%)	F1-Score (%)
*w*/*o* TFME	73.52 ± 2.52	75.54 ± 4.14	66.99 ± 5.06	70.87 ± 2.98
*w*/*o* FSDE	76.38 ± 3.55	73.87 ± 2.92	78.88 ± 7.06	76.19 ± 4.27
*w*/*o* ADTF	80.23 ± 1.00	78.96 ± 3.28	80.88 ± 6.43	79.69 ± 1.86
*w*/*o* BiMamba	77.57 ± 2.17	76.68 ± 2.12	76.84 ± 4.29	76.71 ± 2.61
FSC-BiMamba	82.70 ± 2.37	82.50 ± 1.65	81.36 ± 5.33	81.86 ± 2.90

**Table 8:** Ablation results of FSC-BiMamba on the Mumtaz2016 dataset.

Model	Accuracy (%)	Precision (%)	Recall (%)	F1-Score (%)
*w*/*o* TFME	80.16 ± 11.79	83.00 ± 12.95	81.59 ± 20.99	80.14 ± 12.47
*w*/*o* FSDE	83.01 ± 10.96	85.53 ± 9.85	77.72 ± 21.01	80.68 ± 15.91
*w*/*o* ADTF	83.92 ± 8.79	87.94 ± 7.48	78.25 ± 20.64	81.61 ± 13.47
*w*/*o* BiMamba	77.02 ± 8.29	77.84 ± 8.62	78.10 ± 21.74	76.19 ± 12.70
FSC-BiMamba	84.55 ± 8.01	89.02 ± 6.13	79.73 ± 21.98	82.14 ± 13.92

**Table 9:** Sensitivity analysis of the window duration and the number of windows per sequence.

Window Duration (s)	Number of Windows per Sequence	MODMA Accuracy (%)	MODMA F1-Score (%)	Mumtaz2016 Accuracy (%)	Mumtaz2016 F1-Score (%)
1	8	75.64 ± 4.64	75.32 ± 4.34	82.36 ± 8.57	81.29 ± 11.09
2	8	**82.70 ± 2.37**	**81.86 ± 2.90**	**84.55 ± 8.01**	**82.14 ± 13.92**
4	8	79.13 ± 2.30	78.60 ± 3.27	81.52 ± 10.52	79.34 ± 14.84
2	4	81.93 ± 1.15	80.37 ± 1.14	84.40 ± 9.41	81.88 ± 15.02
2	16	79.15 ± 3.31	80.89 ± 10.85	82.86 ± 8.76	80.55 ± 14.51

Bold values indicate the highest mean performance for each metric at each evaluation level.

## Data Availability

The data analyzed in this study were derived from publicly available resources. The MODMA dataset is available at https://modma.lzu.edu.cn/data/index (accessed on 10 July 2026), and the Mumtaz2016 dataset is available at https://figshare.com/articles/dataset/EEG_Data_New/4244171 (accessed on 10 July 2026).
